# Supplementary material for: QuPath: Open source software for digital pathology image analysis
Source: Sci Rep. 2017 Dec 4;7:16878. doi: 10.1038/s41598-017-17204-5 (PMC5715110; doi:10.1038/s41598-017-17204-5)
Supplement: Supplementary file 1 — Supplementary Information [file 41598_2017_17204_MOESM1_ESM.pdf]

Supplementary Information

QuPath: Open source software for digital pathology image analysis

Authors:

Peter Bankhead[1], Maurice B Loughrey[1,2], José A Fernández[1], Yvonne Dombrowski[3], Darragh G McArt[1], Philip D Dunne[1], Stephen McQuaid[1,2], Ronan T Gray[4], Liam J Murray[4], Helen G Coleman[4], Jacqueline A James[1,2], Manuel Salto-Tellez[1,2,*], Peter W Hamilton[1,*]

Affiliations:

[1] Northern Ireland Molecular Pathology Laboratory, Centre for Cancer Research and Cell Biology, Queen's University Belfast, Belfast, Northern Ireland, UK

[2] Tissue Pathology, Belfast Health and Social Care Trust, Belfast, Northern Ireland, Northern Ireland, UK

[3] Centre for Experimental Medicine, Queen's University Belfast, Belfast, Northern Ireland, UK

[4] Cancer Epidemiology and Health Services Research Group, Centre for Public Health, Queen's University Belfast, Belfast, Northern Ireland, UK

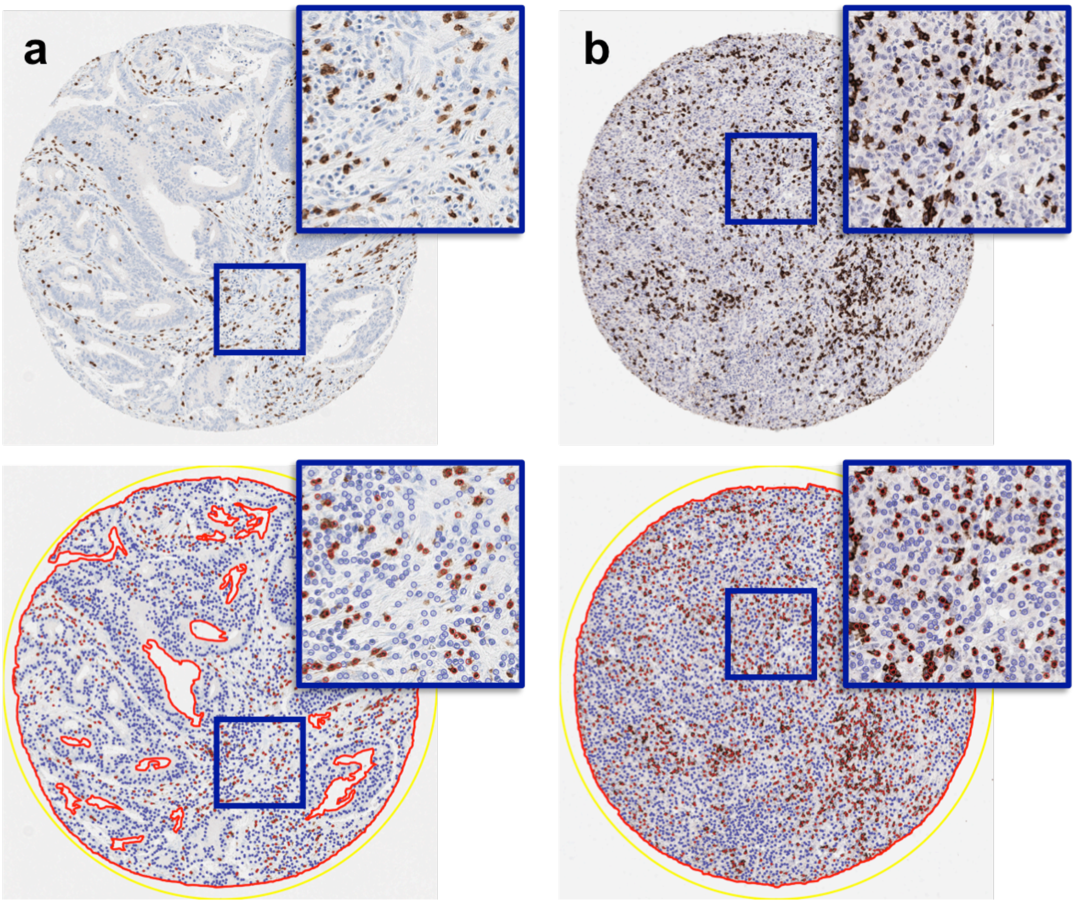

Supplementary Figure 1

Analysis of (**a**) CD3 and (**b**) CD8 as the number of positive cells per mm^2 tissue. The red boundary in each markup image denotes the detected tissue region. Detected positive cells are shown as small red circles. The total count of positive cells is divided by the tissue area to give the final output. Negative cells (blue) are detected, but do not contribute to the score.

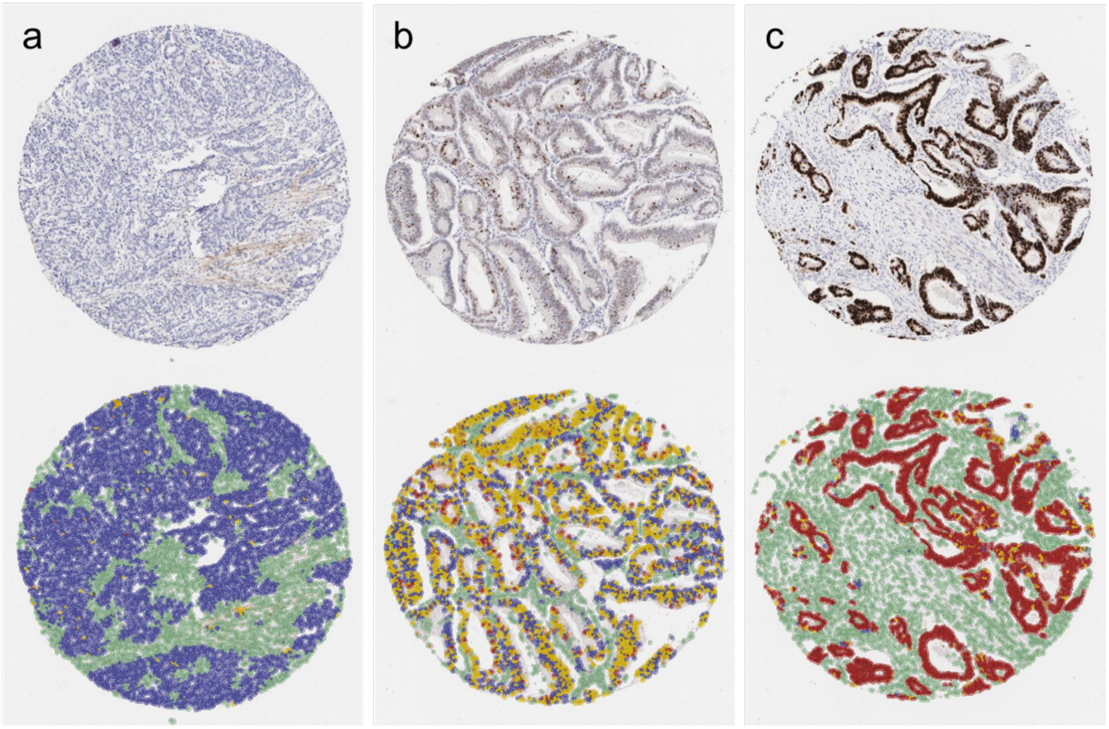

Supplementary Figure 2

Original and markup images of p53-stained TMA cores showing cell detection and classification for p53 immunoscoring using QuPath. Non-epithelial cells are shown in light green. Epithelial cells are colored according to p53 expression based on mean DAB nuclear staining intensity as follows: blue (negative), yellow (weak positive), orange (moderate positive) and red (strong positive). (**a**) Extreme negative p53 expression (H-score = 1.4). (**b**) Moderate p53 expression (H-score = 74.7). (**c**) Extreme positive p53 expression (H-score = 277.3).

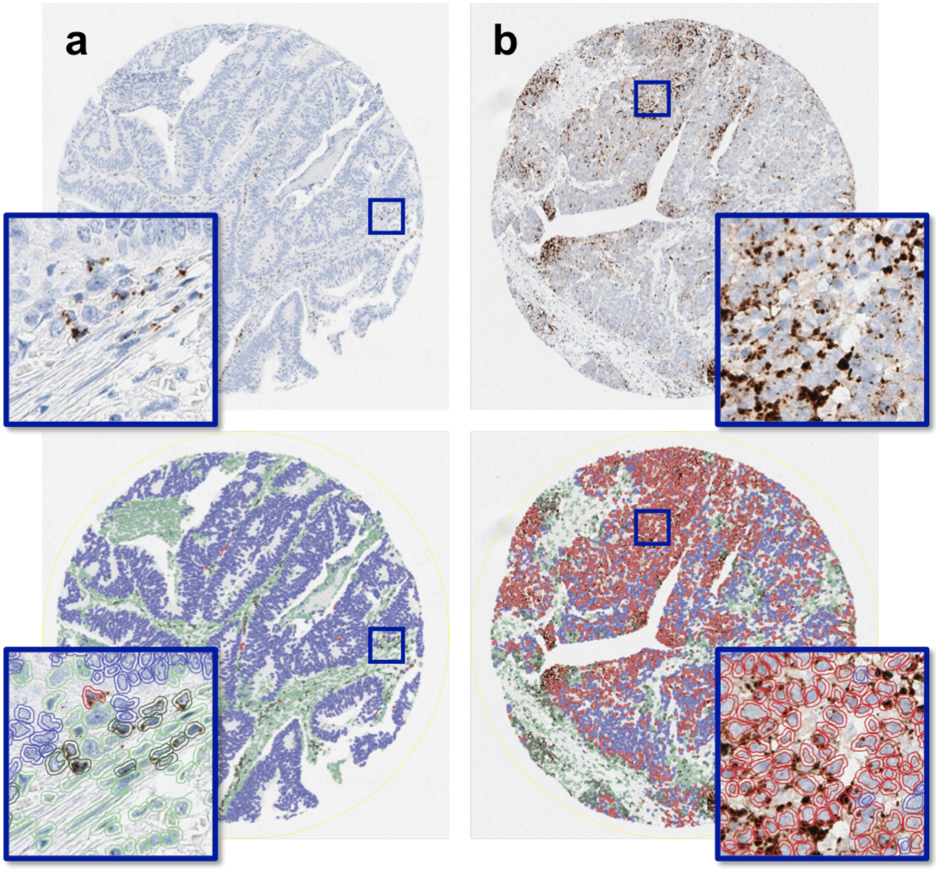

Supplementary Figure 3

Original and markup images showing PD-L1 cell detection and classification using QuPath. Detected cells are color-coded according to classification: light green (non-epithelial, negative), dark green (non-epithelial, positive), blue (epithelial, negative) and red (epithelial, positive). (**a**) Moderate PD-L1 expression (2.9 % positive cells). (**b**) High PD-L1 expression (48.7 % positive cells).

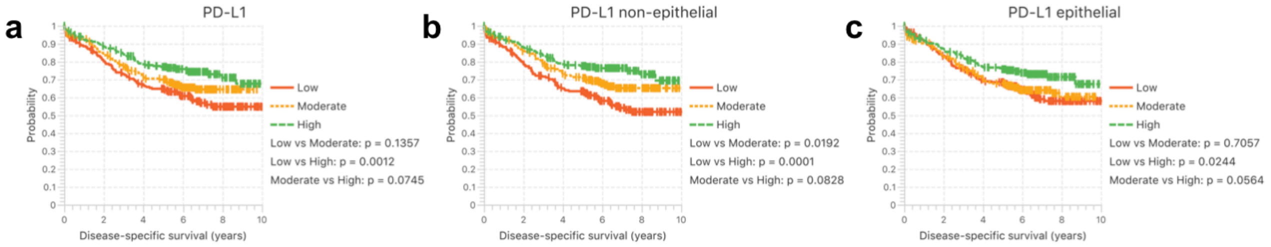

Supplementary Figure 4

Survival analysis of PD-L1 based on tertiles. (**a**) A moderate dose-response effect is suggested when considering PD-L1 in all cells. Tertile cutoff values are 0.8% and 2.6% positive cells per core. (**b**) Considering only cells classified as non-epithelial, a more pronounced separation in survival curves is seen. Cutoff values are also increased to 1.4% and 4.3%, indicating that most cells expressing PD-L1 positivity are non-epithelial. (**c**) A decreased separation in survival curves is seen when considering PD-L1 expression only within cells classified as epithelial. A statistically significant separation between low-and high expression is still evident, but this is based on extremely low cutoff values of 0.23% and 0.85% positive cells, and should therefore be interpreted with caution.

Batch processing scripts

This file contains batch processing scripts used in the analysis described in Bankhead et al. 'QuPath: Open source software for digital pathology image analysis'.

It is not intended that these scripts would be used directly for other projects with similar goals (e.g. scoring CD3 or PD-L1 in tissue microarrays), since differences in antibodies, staining and scanning mean that the parameters required for each dataset may be quite different. Nevertheless, the scripts may be used as a template for how similar analysis may be performed using QuPath.

The majority of the scripts here were generated automatically from QuPath's 'Command history' while interactively analyzing an image, with minor editing afterwards to remove any unnecessary steps applied when running commands multiple times to tune parameters.

Further documentation describing how to work with QuPath is provided at https://github.com/qupath/qupath/wiki

After export, tissue microarray results can be viewed with QuPath's 'TMA data viewer' or imported into other software.

CD3 & CD8 scripts

These scripts calculate positive cell counts and densities for CD3 and CD8 Tissue Microarrays, as described in Bankhead et al. 'QuPath: Open source software for digital pathology image analysis'.

It is assumed that the slides have already been dearrayed (e.g. using QuPath's 'TMA -> TMA dearrayer' command).

Each script automates applying the results of running the following commands:
* 'Analyze -> Preprocessing -> Estimate stain vectors'
* 'Analyze -> Preprocessing -> Simple tissue detection'
* 'Analyze -> Cell analysis -> Fast cell counts (brightfield)'
* 'File -> Export TMA data'

For the first three, it is strongly advised to apply these commands directly to a representative image in order to determine appropriate parameters, which will depend upon the specific scanner and staining used.

QuPath logs the parameters that were used, and can generate the appropriate lines of code to use within the script.

Further documentation describing how to do this is available at https://github.com/qupath/qupath/wiki

CD3 positive cell densities CRC.groovy

```
/*
 * CD3 positive cell detections using QuPath.
 *
 * @author Pete Bankhead
 */

// Set the color deconvolution stains for this image set
// (with the help of the 'Estimate stain vectors' command)
setColorDeconvolutionStains('{"Name" : "H-DAB CD3", "Stain 1" : "Hematoxylin", "Values 1" : "0.71778 0.61619 0.32420", "Stain 2" : "DAB",
"Values 2" : "0.26484 0.55793 0.78649", "Background" : "[242.0, 243.0, 242.0]"}');

// Detect tissue in each TMA core using 'Simple tissue detection' command
selectTMACores()
runPlugin('qupath.imagej.detect.tissue.SimpleTissueDetection2', '{"threshold": 238,  "requestedPixelSizeMicrons": 2,  "minAreaMicrons":
100000.0,  "maxHoleAreaMicrons": 1000.0,  "darkBackground": false,  "smoothImage": true,  "medianCleanup": true,  "dilateBoundaries": false,
"smoothCoordinates": true,  "excludeOnBoundary": false,  "singleAnnotation": true}');

// Detect positive (and negative) cells by counting smoothed peaks with 'Fast cell counts (brightfield)' command
selectAnnotations();
runPlugin('qupath.opencv.CellCountsCV', '{"stainChannel": "Hematoxylin",  "gaussianSigmaMicrons": 1.75,  "backgroundRadiusMicrons": 15.0,
"doDoG": false,  "threshold": 0.1,  "thresholdDAB": 0.2,  "detectionDiameter": 25.0}');

// Export summary results
def exportPath = buildFilePath(PROJECT_BASE_DIR, 'results', 'CD3 final')
mkdirs(exportPath)
exportTMAData(exportPath, 8)
```

CD8 positive cell densities CRC.groovy

```
/*
 * CD8 positive cell detections using QuPath.
 *
 * @author Pete Bankhead
 */

// Set the color deconvolution stains for this image set
// (with the help of the 'Estimate stain vectors' command)
setColorDeconvolutionStains('{"Name" : "H-DAB CD8", "Stain 1" : "Hematoxylin", "Values 1" : "0.69501 0.62707 0.35178", "Stain 2" : "DAB",
"Values 2" : "0.36710 0.61816 0.69506", "Background" : "[244.0, 244.0, 244.0]"}');

// Detect tissue in each TMA core using 'Simple tissue detection' command
selectTMACores()
runPlugin('qupath.imagej.detect.tissue.SimpleTissueDetection2', '{"threshold": 238,  "requestedPixelSizeMicrons": 2,  "minAreaMicrons":
100000.0,  "maxHoleAreaMicrons": 1000.0,  "darkBackground": false,  "smoothImage": true,  "medianCleanup": true,  "dilateBoundaries": false,
"smoothCoordinates": true,  "excludeOnBoundary": false,  "singleAnnotation": true}');

// Detect positive (and negative) cells by counting smoothed peaks with 'Fast cell counts (brightfield)' command
selectAnnotations();
runPlugin('qupath.opencv.CellCountsCV', '{"stainChannel": "Hematoxylin + DAB",  "gaussianSigmaMicrons": 2.0,  "backgroundRadiusMicrons": 15.0,
"doDoG": false,  "threshold": 0.15,  "thresholdDAB": 0.3,  "detectionDiameter": 25.0}');

// Export summary results
def exportPath = buildFilePath(PROJECT_BASE_DIR, 'results', 'CD8 final')
mkdirs(exportPath)
exportTMAData(exportPath, 8)
```

p53 scripts

These scripts were used to score p53 within tissue microarrays (TMAs), as described in Bankhead et al. 'QuPath: Open source software for digital pathology image analysis'.

The process is as follows:

1. Add the relevant slides to a QuPath project.

2. Run 'p53_preprocessing.groovy' across all slides in the project.

3. Check visually whether the dearraying has been successful, and amend if required (e.g. to relocate cores, or exclude cores exhibiting prominent artifacts). Optionally import patient identifiers for each slide if required (with 'File -> Import TMA map').

4. Run 'p53_preprocessing.groovy' a second time to detect cells within each TMA core.

5. Use 'Classify -> Create detection classifier' to interactively train a classifier to identify tumor cells, and set intensity thresholds to classify tumor cells according to mean DAB staining intensity within the nucleus.

6. Save the classifier as 'classifiers/p53.qpclassifier' inside the project directory.

7. Run 'p53_classify_and_export.groovy' to classify all cells and export the results.

All scripts should be run in batch over all images.

Further documentation describing the use of QuPath is available at https://github.com/qupath/qupath/wiki

p53_preprocessing.groovy

```groovy
/**
 * Script to set the stains for a brightfield tissue microarray (TMA) slide stained with hematoxylin & DAB,
 * then dearray it, detect cells within each core, and compute additional features per cell.
 *
 * Note: this script is typically run twice: first to dearray the slide, and then later to detect the cells.
 * Between runs, the quality of the dearraying should be checked, and any required patient identifiers imported.
 *
 * @author Pete Bankhead
 */

// Ensure the image type is set to brightfield, with hematoxylin & DAB staining
setImageType('BRIGHTFIELD_H_DAB');

// Set color deconvolution stains for the current image (with the help of the 'Estimate stain vectors' command
setColorDeconvolutionStains('{"Name" : "H-DAB p53", "Stain 1" : "Hematoxylin", "Values 1" : "0.68393 0.64345 0.34381", "Stain 2" : "DAB",
"Values 2" : "0.34404 0.57141 0.74507", "Background" : "[242.0, 243.0, 242.0]"}');

// If this slide isn't already dearrayed, then dearray it and return -
// the dearraying should be checked (and, if necessary, refined) before continuing with any processing.
if (!isTMADearrayed()) {
   runPlugin('qupath.imagej.detect.dearray.TMADearrayerPluginIJ', '{"coreDiameterMM": 1.2,  "labelsHorizontal": "1-16",  "labelsVertical": "A-
J",  "labelOrder": "Row first",  "densityThreshold": 5,  "boundsScale": 105}');
   return;
}

// If the slide has been dearrayed, then select all TMA cores and run cell detection
selectTMACores();
runPlugin('qupath.imagej.detect.nuclei.PositiveCellDetection', '{"detectionImageBrightfield": "Hematoxylin OD",  "requestedPixelSizeMicrons":
0.5,  "backgroundRadiusMicrons": 8.0,  "medianRadiusMicrons": 0.0,  "sigmaMicrons": 1.5,  "minAreaMicrons": 10.0,  "maxAreaMicrons": 400.0,
"threshold": 0.1,  "maxBackground": 2.0,  "watershedPostProcess": true,  "excludeDAB": false,  "cellExpansionMicrons": 5.0,  "includeNuclei":
true,  "smoothBoundaries": true,  "makeMeasurements": true,  "thresholdCompartment": "Nucleus: DAB OD mean",  "thresholdPositive1": 0.15,
"thresholdPositive2": 0.3,  "thresholdPositive3": 0.6,  "singleThreshold": false}');

// Remove unnecessary or highly-correlated measurements
// (This isn't essential, but may improve the performance of the cell classifier)
removeMeasurements(qupath.lib.objects.PathCellObject, "Nucleus: Hematoxylin OD range", "Cell: Circularity", "Cytoplasm: DAB OD max", "Cell:
Eccentricity", "Nucleus: DAB OD max", "Cell: DAB OD min", "Nucleus: Hematoxylin OD max", "Cell: Max caliper", "Cell: DAB OD std dev", "Cell:
Perimeter", "Nucleus: DAB OD range", "Cytoplasm: DAB OD std dev", "Nucleus: Hematoxylin OD min", "Cytoplasm: DAB OD min", "Cell: Min caliper",
"Cell: DAB OD max", "Nucleus: DAB OD min");

// Apply smoothing to supplement the features for each cell with weighted sums of the features from neighboring cells
runPlugin('qupath.lib.plugins.objects.SmoothFeaturesPlugin', '{"fwhmMicrons": 25.0,  "smoothWithinClasses": false,  "useLegacyNames": true}');
runPlugin('qupath.lib.plugins.objects.SmoothFeaturesPlugin', '{"fwhmMicrons": 75.0,  "smoothWithinClasses": false,  "useLegacyNames": true}');
```

p53_classify_and_export.groovy

```
/**
 * Script to run a classifier over a TMA image set, and export the results.
 *
 * @author Pete Bankhead
 */

// Load and run a trained classifier
def pathClassifier = buildFilePath(PROJECT_BASE_DIR, 'classifiers', 'p53.qpclassifier')
runClassifier(pathClassifier);

// Export the results
def pathExport = buildFilePath(PROJECT_BASE_DIR, 'results', 'p53 classified-final')
mkdirs(pathExport)
exportTMAData(pathExport, 8)
```

PD-L1 scripts

These scripts were used to score PD-L1 within tissue microarrays (TMAs), as described in Bankhead et al. 'QuPath: Open source software for digital pathology image analysis'.

The process is as follows:

1. Add the relevant slides to a QuPath project.

2. Run 'PDL1_preprocessing.groovy' across all slides in the project.

3. Check visually whether the dearraying has been successful, and amend if required (e.g. to relocate cores, or exclude cores exhibiting prominent artifacts). Optionally import patient identifiers for each slide if required (with 'File -> Import TMA map').

4. Run 'PDL1_preprocessing.groovy' a second time to detect cells within each TMA core.

5. Use 'Classify -> Create detection classifier' to interactively train a classifier to identify tumor cells, and set intensity thresholds to classify tumor cells according to maximum DAB staining intensity within the cell (or optionally another measurement, if required).

6. Save the classifier as 'classifiers/PDL1.qpclassifier' inside the project directory.

7. Run 'PDL1_classify_and_export.groovy' to classify all cells and export the results.

All scripts should be run in batch over all images.

Further documentation describing the use of QuPath is available at https://github.com/qupath/qupath/wiki

PDL1_preprocessing.groovy

```groovy
/**
 * Script to set the stains for a brightfield tissue microarray (TMA) slide stained with hematoxylin & DAB,
 * then dearray it, detect cells within each core, and compute additional features per cell.
 *
 * Note: this script is typically run twice: first to dearray the slide, and then later to detect the cells.
 * Between runs, the quality of the dearraying should be checked, and any required patient identifiers imported.
 *
 * @author Pete Bankhead
 */

// Ensure the image type is set to brightfield, with hematoxylin & DAB staining
setImageType('BRIGHTFIELD_H_DAB');

// Set color deconvolution stains for the current image (with the help of the 'Estimate stain vectors' command
setColorDeconvolutionStains('{"Name" : "H-DAB PDL1", "Stain 1" : "Hematoxylin", "Values 1" : "0.74072 0.58133 0.33672", "Stain 2" : "DAB",
"Values 2" : "0.31571 0.55024 0.77302", "Background" : "[242.0, 242.0, 242.0]"}');

// If this slide isn't already dearrayed, then dearray it and return -
// the dearraying should be checked (and, if necessary, refined) before continuing with any processing.
if (!isTMADearrayed()) {
   runPlugin('qupath.imagej.detect.dearray.TMADearrayerPluginIJ', '{"coreDiameterMM": 1.2,  "labelsHorizontal": "1-18",  "labelsVertical": "A-
M",  "labelOrder": "Row first",  "densityThreshold": 10,  "boundsScale": 105}');
   return;
}

// If the slide has been dearrayed, then select all TMA cores and run cell detection
selectTMACores();
runPlugin('qupath.imagej.detect.nuclei.PositiveCellDetection', '{"detectionImageBrightfield": "Hematoxylin OD",  "requestedPixelSizeMicrons":
0.5,  "backgroundRadiusMicrons": 0.0,  "medianRadiusMicrons": 0.0,  "sigmaMicrons": 1.5,  "minAreaMicrons": 10.0,  "maxAreaMicrons": 400.0,
"threshold": 0.1,  "maxBackground": 2.0,  "watershedPostProcess": true,  "excludeDAB": true,  "cellExpansionMicrons": 2.0,  "includeNuclei":
true,  "smoothBoundaries": true,  "makeMeasurements": true,  "thresholdCompartment": "Cell: DAB OD max",  "thresholdPositive1": 0.5,
"thresholdPositive2": 0.4,  "thresholdPositive3": 0.6,  "singleThreshold": true}');

// Remove unnecessary or highly-correlated measurements
// (This isn't essential, but may improve the performance of the cell classifier)
removeMeasurements(qupath.lib.objects.PathCellObject, "Nucleus: Hematoxylin OD range", "Cell: Area", "Cell: Circularity", "Nucleus: Max
caliper", "Cell: Eccentricity", "Delaunay: Mean distance", "Cell: DAB OD min", "Cell: Max caliper", "Cell: DAB OD std dev", "Nucleus: DAB OD
sum", "Cell: Perimeter", "Nucleus: DAB OD range", "Cytoplasm: DAB OD std dev", "Cytoplasm: DAB OD min", "Cell: Min caliper", "Nucleus:
Perimeter", "Nucleus: Min caliper", "Nucleus: DAB OD min");

// Apply smoothing to supplement the features for each cell with weighted sums of the features from neighboring cells
runPlugin('qupath.lib.plugins.objects.SmoothFeaturesPlugin', '{"fwhmMicrons": 25.0,  "smoothWithinClasses": false,  "useLegacyNames": true}');
runPlugin('qupath.lib.plugins.objects.SmoothFeaturesPlugin', '{"fwhmMicrons": 75.0,  "smoothWithinClasses": false,  "useLegacyNames": true}');
```

PDL1_classify_and_export.groovy

```groovy
/**
 * Sub-classify cells within a TMA image based upon a specific intensity feature and threshold,
 * after optionally applying a trained detection classifier (e.g. to distinguish 'Tumor' and 'Stroma' classes)
 *
 * Additionally, export the results if required.
 *
 * @author Pete Bankhead
 */


import qupath.lib.classifiers.PathClassificationLabellingHelper
import qupath.lib.objects.*
import qupath.lib.objects.classes.*
import qupath.lib.objects.classes.PathClassFactory.PathClasses
import qupath.lib.objects.hierarchy.*
import qupath.lib.projects.ProjectImageEntry
import qupath.lib.scripting.QPEx
import qupath.lib.common.ColorTools

//-------------------------------------------------------------------
// Parameters to modify

// The marker is used to determine an export directory
String marker = "PDL1";

// Specify which measurement to use to determine cell positivity
String measurementName = "Cell: DAB OD max";
// Determine a threshold to use to determine cell positivity
double threshold = 0.35;

// Decide whether to run classifier - set to false to only apply intensity (re)classification
doClassify = false;
// Specify the classifier path; only used if doClassify is true
def classifierPath = buildFilePath(PROJECT_BASE_DIR, 'classifiers', 'PDL1.qpclassifier')

// Decide whether to export the results and images
doExport = true;
// Specify the export path; only used if doExport is true
def exportPath = buildFilePath(PROJECT_BASE_DIR, 'results', marker)

//-------------------------------------------------------------------

// Run a classifier, if required
if (doClassify) {
    print("Running classifier...")
    QPEx.runClassifier(classifierPath);
}
```

```
// Ensure colors set for stroma class (tumor class set automatically)
def stromaClass = PathClassFactory.getPathClass('Stroma')
PathClassFactory.getPositive(stromaClass, null).setColor(ColorTools.makeScaledRGB(stromaClass.getColor(), 0.5))
PathClassFactory.getNegative(stromaClass, null).setColor(ColorTools.makeScaledRGB(stromaClass.getColor(), 1))

// Classify all detection objects according to intensity
PathObjectHierarchy hierarchy = QPEx.getCurrentHierarchy();
for (TMACoreObject core : hierarchy.getTMAGrid().getTMACoreList()) {

    // Loop through all the detection objects in the core
    List<PathObject> pathObjects = hierarchy.getDescendantObjects(core, null, PathDetectionObject.class);
    for (PathObject pathObject : pathObjects) {

        // Get the measurement value for this object
        double value = pathObject.getMeasurementList().getMeasurementValue(measurementName);

        // If we don't have a measurement, just continue
        if (Double.isNaN(value))
            continue;

        // Get the base classification - here, we only classify objects that have a base classification that isn't 'Other'
        // (In this case that means 'Tumor' and 'Stroma' classifications will be subclassified according to intensity)
        PathClass baseClass = pathObject.getPathClass() == null ? null : pathObject.getPathClass().getBaseClass();
        if (baseClass == null || baseClass.getName().equals("Other"))
            continue;

        // Set the classification according to thresholded measurement
        if (value >= threshold)
            pathObject.setPathClass(PathClassFactory.getPositive(baseClass, null));
        else
            pathObject.setPathClass(PathClassFactory.getNegative(baseClass, null));
    }
}

// Fire an update event - important so the user interface will refresh
hierarchy.fireHierarchyChangedEvent(this);

// Export results
if (doExport) {
    mkdirs(exportPath)
    QPEx.exportTMAData(exportPath, 8)
}

// Write something informative to make sure we know the script is over
print("Done!");
```

H&E scripts

These scripts were used to calculate the tumor stromal percentage within whole face sections stained with H&E, as described in Bankhead et al. 'QuPath: Open source software for digital pathology image analysis'.

The process is as follows:

1. Add the relevant slides to a QuPath project.

2. For each slide in the project, draw a single annotation around a representative tumor area.

3. Run 'Estimate_background_values.groovy' to automatically determine appropriate 'white' values, to improve the calculation of optical densities. This is optional, but may be helpful for darker scans; it presupposes background areas are available somewhere within the image.

4. Run 'H&E_superpixels_script.groovy' to compute superpixels and features within the annotated regions.

5. Use 'Classify -> Create detection classifier' to interactively train a classifier to distinguish different classes of region within the image. When complete, apply this the classifier to all images.

6. Run 'Export_H&E_tumor_areas.groovy' to export the results.

All scripts should be run in batch over all images.

Further documentation describing the use of QuPath is available at https://github.com/qupath/qupath/wiki

Estimate_background_values.groovy

```
/**
 * Script to estimate the background value in a brightfield whole slide image.
 *
 * This effectively looks for the circle of a specified radius with the highest
 * mean intensity (summed across all 3 RGB channels), and takes the mean red,
 * green and blue within this circle as the background values.
 *
 * The background values are then set in the ColorDeconvolutionStains object
 * for the ImageData, so that they are used for any optical density calculations.
 *
 * This is implemented with the help of ImageJ (www.imagej.net).
 *
 * @author Pete Bankhead
 */

import ij.plugin.filter.RankFilters
import ij.process.ColorProcessor
import qupath.imagej.images.servers.ImagePlusServerBuilder
import qupath.lib.common.ColorTools
import qupath.lib.regions.RegionRequest
import qupath.lib.scripting.QP


// Radius used for background search, in microns (will be used approximately)
double radiusMicrons = 1000

// Calculate a suitable 'preferred' pixel size
// Keep in mind that downsampling involves a form of smoothing, so we just choose
// a reasonable filter size and then adapt the image resolution accordingly
double radiusPixels = 10
double requestedPixelSizeMicrons = radiusMicrons / radiusPixels

// Get the ImageData & ImageServer
def imageData = QP.getCurrentImageData()
def server = imageData.getServer()

// Check we have the right kind of data
if (!imageData.isBrightfield() || !server.isRGB()) {
    print("ERROR: Only brightfield RGB images can be processed with this script, sorry")
    return
}

// Extract pixel size
double pixelSize = server.getAveragedPixelSizeMicrons()
// Choose a default if we need one (i.e. if the pixel size is missing from the image metadata)
if (Double.isNaN(pixelSize))
    pixelSize = 0.5
```

```
// Figure out suitable downsampling value
double downsample = Math.round(requestedPixelSizeMicrons / pixelSize)

// Get a downsampled version of the image as an ImagePlus (for ImageJ)
def request = RegionRequest.createInstance(server.getPath(), downsample, 0, 0, server.getWidth(), server.getHeight())
def serverIJ = ImagePlusServerBuilder.ensureImagePlusWholeSlideServer(server)
def pathImage = serverIJ.readImagePlusRegion(request)

// Check we have an RGB image (we should at this point)
def imp = pathImage.getImage(false)
def ip = imp.getProcessor()
if (!(ip instanceof ColorProcessor)) {
    print("Sorry, the background can only be set for a ColorProcessor, but the current ImageProcessor is " + ip)
    return
}

// Apply filter
if (ip.getWidth() <= radiusPixels*2 || ip.getHeight() <= radiusPixels*2) {
    print("The image is too small for the requested radius!")
    return
}
new RankFilters().rank(ip, radiusPixels, RankFilters.MEAN)

// Find the location of the maximum across all 3 channels
double maxValue = Double.NEGATIVE_INFINITY
double maxRed = 0
double maxGreen = 0
double maxBlue = 0
for (int y = radiusPixels; y < ip.getHeight()-radiusPixels; y++) {
    for (int x = radiusPixels; x < ip.getWidth()-radiusPixels; x++) {
        int rgb = ip.getPixel(x, y)
        int r = ColorTools.red(rgb)
        int g = ColorTools.green(rgb)
        int b = ColorTools.blue(rgb)
        double sum = r + g + b
        if (sum > maxValue) {
            maxValue = sum
            maxRed = r
            maxGreen = g
            maxBlue = b
        }
    }
}

// Print the result
print("Background RGB values: " + maxRed + ", " + maxGreen + ", " + maxBlue)

// Set the ImageData stains
```

```
def stains = imageData.getColorDeconvolutionStains()
def stains2 = stains.changeMaxValues(maxRed, maxGreen, maxBlue)
imageData.setColorDeconvolutionStains(stains2)
```

H&E_superpixels_script.groovy

```groovy
/*
 * Generate superpixels & compute features for H&E tumor stromal analysis using QuPath.
 *
 * @author Pete Bankhead
 */

// Compute superpixels within annotated regions
selectAnnotations()
runPlugin('qupath.imagej.superpixels.SLICSuperpixelsPlugin', '{"sigmaMicrons": 5.0,  "spacingMicrons": 40.0,  "maxIterations": 10,  
"regularization": 0.25,  "adaptRegularization": false,  "useDeconvolved": false}');

// Add Haralick texture features based on optical densities, and mean Hue for each superpixel
selectDetections();
runPlugin('qupath.lib.algorithms.IntensityFeaturesPlugin', '{"pixelSizeMicrons": 2.0,  "region": "ROI",  "tileSizeMicrons": 25.0,  "colorOD": 
true,  "colorStain1": false,  "colorStain2": false,  "colorStain3": false,  "colorRed": false,  "colorGreen": false,  "colorBlue": false,  
"colorHue": true,  "colorSaturation": false,  "colorBrightness": false,  "doMean": true,  "doStdDev": false,  "doMinMax": false,  "doMedian": 
false,  "doHaralick": true,  "haralickDistance": 1,  "haralickBins": 32}');

// Add smoothed measurements to each superpixel
selectAnnotations();
runPlugin('qupath.lib.plugins.objects.SmoothFeaturesPlugin', '{"fwhmMicrons": 50.0,  "smoothWithinClasses": false,  "useLegacyNames": 
false}');
```

Export_H&E_tumor_areas.groovy

```groovy
/**
 * Script to compute areas for detection objects with different classifications.
 *
 * Primarily useful for converting classified superpixels into areas.
 *
 * @author Pete Bankhead
 */


import qupath.lib.common.GeneralTools
import qupath.lib.gui.QuPathGUI
import qupath.lib.images.servers.ImageServer
import qupath.lib.objects.PathDetectionObject
import qupath.lib.objects.PathObject
import qupath.lib.objects.classes.PathClass
import qupath.lib.objects.classes.PathClassFactory
import qupath.lib.objects.hierarchy.PathObjectHierarchy
import qupath.lib.roi.interfaces.PathArea
import qupath.lib.roi.interfaces.ROI
import qupath.lib.scripting.QPEx
import qupath.lib.gui.ImageWriterTools
import qupath.lib.regions.RegionRequest

import java.awt.image.BufferedImage

//-----------------------------------------------------------------------------

// If 'exportImages' is true, overview images will be written to an 'export' subdirectory
// of the current project, otherwise no images will be exported
boolean exportImages = true

// Define which classes to analyze, in terms of determining areas etc.
def classesToAnalyze = ["Tumor", "Stroma", "Other"]

// Format output 3 decimal places
int nDecimalPlaces = 3

// If the patient ID is encoded in the filename, a closure defined here can parse it
// (By default, this simply strips off anything after the final dot - assumed to be the file extension)
def parseUniqueIDFromFilename = { n ->
    int dotInd = n.lastIndexOf('.')
    if (dotInd < 0)
        return n
    return n.substring(0, dotInd)
}

// The following closure handled the specific naming scheme used for the images in the paper
```

```
// Uncomment to apply this, rather than the default method above
//parseUniqueIDFromFilename = {n ->
//    def uniqueID = n.trim()
//    if (uniqueID.charAt(3) == '-')
//        uniqueID = uniqueID.substring(0, 3) + uniqueID.substring(4)
//    return uniqueID.split("[-. ]")[0]
//    }

//-----------------------------------------------------------------------------

// Convert class name to PathClass objects
Set<PathClass> pathClasses = new TreeSet<>()
for (String className : classesToAnalyze) {
    pathClasses.add(PathClassFactory.getPathClass(className))
}

// Get a formatter
// Note: to run this script in earlier versions of QuPath,
// change createFormatter to getFormatter
def formatter = GeneralTools.createFormatter(nDecimalPlaces)

// Get access to the current ImageServer - required for pixel size information
ImageServer<?> server = QPEx.getCurrentImageData().getServer()
double pxWidthMM = server.getPixelWidthMicrons() / 1000
double pxHeightMM = server.getPixelHeightMicrons() / 1000

// Get access to the current hierarchy
PathObjectHierarchy hierarchy = QPEx.getCurrentHierarchy()

// Loop through detection objects (here, superpixels) and increment total area calculations for each class
Map<PathClass, Double> areaMap = new TreeMap<>()
double areaTotalMM = 0
for (PathObject tile : hierarchy.getObjects(null, PathDetectionObject.class)) {

    // Check the classification
    PathClass pathClass = tile.getPathClass()
    if (pathClass == null)
        continue

    // Add current area
    ROI roi = tile.getROI()
    if (roi instanceof PathArea) {
        double area = ((PathArea)roi).getScaledArea(pxWidthMM , pxHeightMM)
        areaTotalMM += area
        if (areaMap.containsKey(pathClass))
            areaMap.put(pathClass, area + areaMap.get(pathClass))
        else
            areaMap.put(pathClass, area)
```

```
    }

}

// Loop through each classification & prepare output
double areaSumMM = 0
double areaTumor = 0
double areaStroma = 0

// Include image name & ID
String delimiter = "\t"
StringBuilder sbHeadings = new StringBuilder("Image").append(delimiter).append("Unique ID")
String uniqueID = parseUniqueIDFromFilename(server.getShortServerName())
StringBuilder sb = new StringBuilder(server.getShortServerName()).append(delimiter).append(uniqueID)

// Compute areas per class
for (PathClass pathClass : pathClasses) {

    // Extract area from map - or zero, if it does not occur in the map
    double area = areaMap.containsKey(pathClass) ? areaMap.get(pathClass) : 0

    // Update total & record tumor area, if required
    areaSumMM += area
    if (pathClass.getName().toLowerCase().contains("tumor") || pathClass.getName().toLowerCase().contains("tumour"))
        areaTumor += area
    if (pathClass.getName().toLowerCase().contains("stroma"))
        areaStroma += area

    // Display area for classification
    sbHeadings.append(delimiter).append(pathClass.getName()).append(" Area mm^2")
    sb.append(delimiter).append(formatter.format(area))
}

// Append the total area
sbHeadings.append(delimiter).append("Total area mm^2")
sb.append(delimiter).append(formatter.format(areaTotalMM))

// Append the calculated stromal percentage
sbHeadings.append(delimiter).append("Stromal percentage")
sb.append(delimiter).append(formatter.format(areaStroma / (areaTumor + areaStroma) * 100.0))

// Export images in a project sub-directory, if required
if (exportImages) {

    // Create the directory, if required
    def path = QPEx.buildFilePath(QPEx.PROJECT_BASE_DIR, "export")
    QPEx.mkdirs(path)

    // We need to get the display settings (colors, line thicknesses, opacity etc.) from the current viewer
```

```
    def overlayOptions = QuPathGUI.getInstance().getViewer().getOverlayOptions()
    def imageData = QPEx.getCurrentImageData()
    def name = uniqueID

    // Aim for an output resolution of approx 20 µm/pixel
    double requestedPixelSize = 20
    double downsample = requestedPixelSize / server.getAveragedPixelSizeMicrons()
    RegionRequest request = RegionRequest.createInstance(imageData.getServerPath(), downsample, 0, 0, server.getWidth(), server.getHeight())

    // Write output image, with and without overlay
    def dir = new File(path)
    def fileImage = new File(dir, name + ".jpg")
    BufferedImage img = ImageWriterTools.writeImageRegion(imageData.getServer(), request, fileImage.getAbsolutePath())
    def fileImageWithOverlay = new File(dir, name + "-overlay.jpg")
    ImageWriterTools.writeImageRegionWithOverlay(img, imageData, overlayOptions, request, fileImageWithOverlay.getAbsolutePath())
}

// Write header to output file if it's empty, and print on screen
sbHeadings.append("\n")
sb.append("\n")
def fileOutput = new File(QPEx.buildFilePath(QPEx.PROJECT_BASE_DIR, "Results.txt"))
if (fileOutput.length() == 0) {
    print(sbHeadings.toString())
    fileOutput << sbHeadings.toString()
}

// Write data to output file & print on screen
fileOutput << sb.toString()
print(sb.toString())
```
